# Maternal high-fat diet induces sex-specific changes to glucocorticoid and inflammatory signaling in response to corticosterone and lipopolysaccharide challenge in adult rat offspring

**DOI:** 10.1186/s12974-020-01798-1

**Published:** 2020-04-15

**Authors:** Sanoji Wijenayake, Mouly F. Rahman, Christine M. W. Lum, Wilfred C. De Vega, Aya Sasaki, Patrick O. McGowan

**Affiliations:** 1 Center for Environmental Epigenetics and Development, Department of Biological Sciences, University of Toronto, Scarborough, 1265 Military Trail, Toronto, ON Canada; 2 Department of Cell and Systems Biology, University of Toronto, Toronto, ON Canada; 3 Department of Psychology, Department of Physiology, University of Toronto, Toronto, ON Canada

**Keywords:** Maternal high-fat diet, Maternal obesity, Offspring, Neuroinflammation, Glucocorticoid signaling, Transcript response, Corticosterone, Lipopolysaccharide

## Abstract

**Background:**

Maternal obesity as a result of high levels of saturated fat (HFD) consumption leads to significant negative health outcomes in both mother and exposed offspring. Offspring exposed to maternal HFD show sex-specific alterations in metabolic, behavioral, and endocrine function, as well as increased levels of basal neuroinflammation that persists into adulthood. There is evidence that psychosocial stress or exogenous administration of corticosterone (CORT) potentiate inflammatory gene expression; however, the response to acute CORT or immune challenge in adult offspring exposed to maternal HFD during perinatal life is unknown. We hypothesize that adult rat offspring exposed to maternal HFD would show enhanced pro-inflammatory gene expression in response to acute administration of CORT and lipopolysaccharide (LPS) compared to control animals, as a result of elevated basal pro-inflammatory gene expression. To test this, we examined the effects of acute CORT and/or LPS exposure on pro and anti-inflammatory neural gene expression in adult offspring (male and female) with perinatal exposure to a HFD or a control house-chow diet (CHD).

**Methods:**

Rat dams consumed HFD or CHD for four weeks prior to mating, during gestation, and throughout lactation. All male and female offspring were weaned on to CHD. In adulthood, offspring were ‘challenged’ with administration of exogenous CORT and/or LPS, and quantitative PCR was used to measure transcript abundance of glucocorticoid receptors and downstream inflammatory markers in the amygdala, hippocampus, and prefrontal cortex.

**Results:**

In response to CORT alone, male HFD offspring showed increased levels of anti-inflammatory transcripts, whereas in response to LPS alone, female HFD offspring showed increased levels of pro-inflammatory transcripts. In addition, male HFD offspring showed greater pro-inflammatory gene expression and female HFD offspring exhibited increased anti-inflammatory gene expression in response to simultaneous CORT and LPS administration.

**Conclusions:**

These findings suggest that exposure to maternal HFD leads to sex-specific changes that may alter inflammatory responses in the brain, possibly as an adaptive response to basal neuroinflammation.

## Introduction

The hypothalamic-pituitary-adrenal (HPA) axis regulates circulating glucocorticoid (GC) levels at baseline and during stress. In the brain, GC binding to glucocorticoid receptors (GRs) in the amygdala, hippocampus (HPC), and prefrontal cortex (PFC) send feedback signals to the hypothalamus to mediate HPA axis activation and inhibition [1, 2]. GRs also mediate inflammatory signalling in the brain. GC-GR binding promotes the expression of anti-inflammatory genes, including mitogen-activated protein kinase phosphatase 1 (MKP1) and NFκB-inhibitor alpha (IκBα). MKP1 and IκBα have been shown to inhibit nuclear translocation of nuclear factor kappa beta (NFκB) and thereby reduce NFκB-mediated transcription of pro-inflammatory cytokines, including IL-6 [3, 4]. While physiological levels of corticosterone (CORT), a well-characterized GC, suppress inflammation, acute increases in CORT potentiate pro-inflammatory processes [3, 5–8]. For example, chronic unpredictable stress potentiates lipopolysaccharide (LPS)-induced NFκB activation and pro-inflammatory cytokine expression in the frontal cortex and HPC. Likewise, increased levels of circulating CORT is associated with a potentiation of LPS-induced pro-inflammatory signalling in the brain [3]. GR antagonism by RU-486 has also been found to blunt the potentiating effect of CORT-mediated stress on LPS-induced pro-inflammation in the frontal cortex and HPC [3, 4, 9]. These findings indicate a positive correlation between CORT-GR binding and pro-inflammation. However, it is unknown how chronic alterations in HPA axis activity due to perinatal HFD exposure may influence neuroinflammatory responses to immune stress and/or acute physiological stress (elevated CORT) later in life.

Free CORT, upon entering the brain, binds to mineralocorticoid receptors (MR). Despite sharing protein domain homology with GR, MR has different functional roles in modulating inflammation in a manner that is not as well understood [10]. CORT affinity for MR is ~ 10-fold higher than for GR at basal levels and is heavily occupied by basal CORT during chronic stress. On the other hand, GR is typically bound by elevated levels of CORT during acute stress [4, 11], but there is some evidence to indicate that MR can also induce fast-acting CORT effects in the HPC [12]. Furthermore, activation of other stress kinase pathways by MR has been linked with NFκB-mediated pro-inflammatory signalling [13, 14]. In contrast, other studies have shown a link between MR and *trans*-repression of NFκB-mediated pro-inflammation, similar to anti-inflammatory roles of GR, albeit weaker [10, 15]. Therefore, it is important to understand the complexities of GR and MR modulation of pro/anti-neuroinflammation not only at basal levels but also in response to acute physiological and immune stressors.

Several studies have linked developmental exposure to high levels of saturated fat through the maternal diet with altered glucocorticoid signalling and HPA axis programming in adult offspring. In particular, high levels of saturated fats in maternal diet induce pro-inflammation in the mother that reaches the developing offspring during gestation and early postnatal life through milk transfer [16–19]. In humans, maternal high-fat diet (HFD) is linked to metabolic disorders and anxiety in children [20–26]. In rodents, offspring born to obese dams, exhibit altered levels of GR and inflammatory genes in brain regions that regulate the HPA axis [27–33]. For example, our lab found increases in NFκB and IL6 transcript levels in the amygdala of females in basal conditions, along with decreased levels of CORT in serum of both sexes [27]. Therefore, in this study, we hypothesized that adult rat offspring exposed to maternal HFD would show enhanced pro-inflammatory gene expression in response to acute administration of CORT and LPS compared to control animals, as a result of elevated levels of basal pro-inflammatory gene expression. Our primary objective was to investigate how maternal HFD exposure during perinatal life impacts corticosterone receptor signalling (GR and MR) and inflammatory gene expression in conditions of stress in adult offspring. To investigate, female and male offspring exposed to maternal CHD or HFD during the perinatal period were administered exogenous CORT to simulate psychological stress, LPS to induce immune stress, or simultaneous CORT and LPS challenge in adulthood. Transcript abundance of CORT receptors, including GR, MR, NFκB, IκBα, and downstream inflammatory genes, were measured in the amygdala, HPC, and PFC. Female and male offspring were examined separately due to prominent sex differences in body weight, endocrine, and behavioral responses to maternal HFD, CORT, and LPS exposures reported previously [27, 30, 34–36].

## Materials and methods

### Animal care

All experimental protocols were approved by the Local Animal Care Committee at the University of Toronto Scarborough and were in accordance with the guidelines of the Canadian Council on Animal Care. The adult rat offspring used for this study were untested littermates from the same cohort of animals used in previous studies published by our group [27, 28]. For breeding, 7-week-old periadolescent male and female Long Evans rats were purchased from Charles River Canada (St. Constant, QC) and housed with same-sex pairs, and maintained on a 12:12 h light/dark cycle (lights turned on from 7:00 am to 7:00 pm) with ad libitum access to food and water. One week later, female breeders were placed on either a high-fat diet (HFD, 5.21 kcal/g, *n* = 15) consisting of 60% saturated fat (90.7% lard and 9.3% soybean oil), 20% protein, and 20% carbohydrate (D12492; Research Diets, Inc. New Brunswick, NJ), or a control house-chow diet (CHD, 3.02 kcal/g, *n* = 14) consisting of 13.5% fat, 28.5% protein, and 58% carbohydrate (5001; Purine Lab Diets. St. Louis, MO) 4 weeks prior to mating, throughout gestation, lactation, and until weaning.

**Fig. 1:**
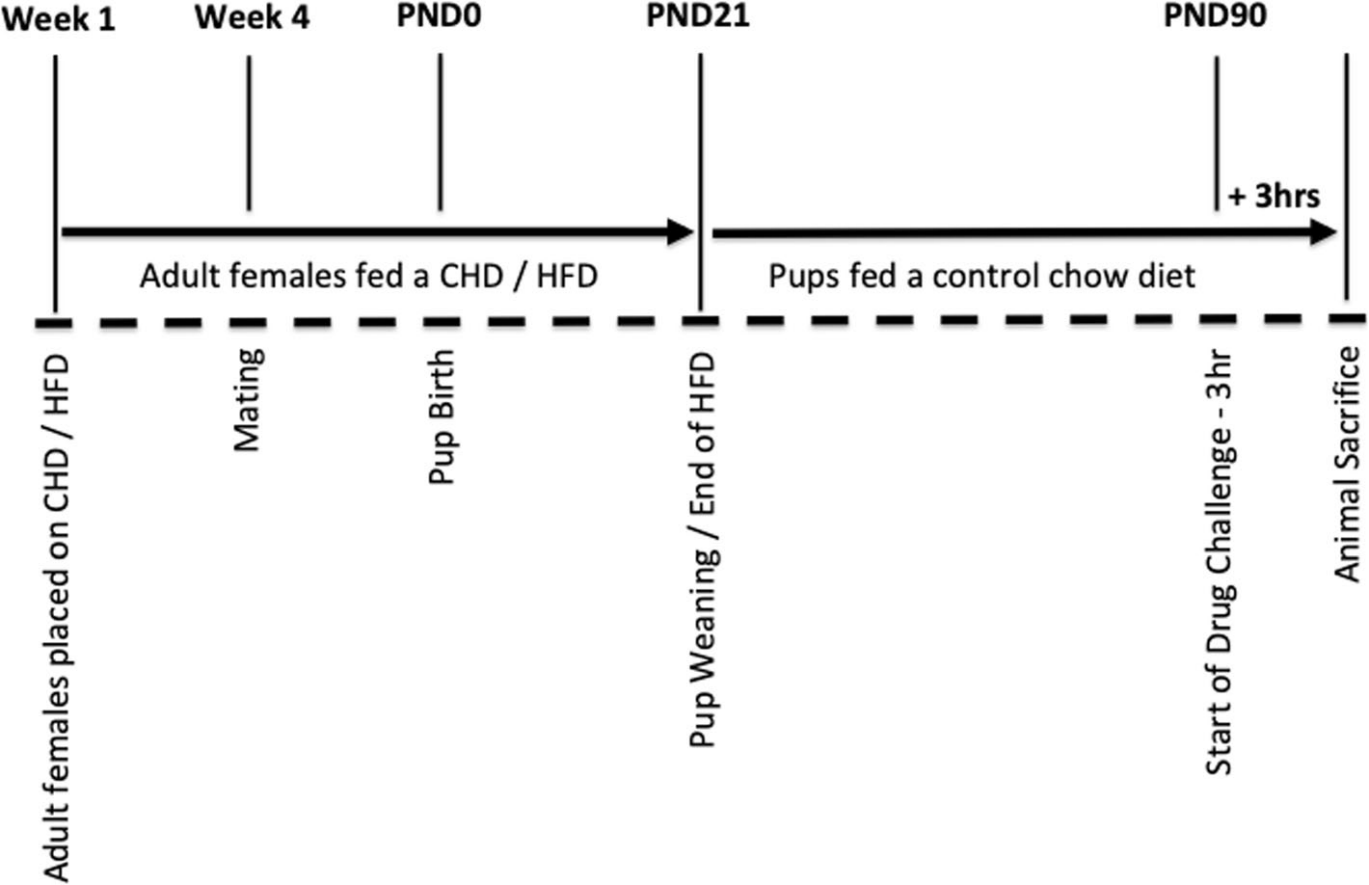
Overview of animal care and stress challenges. Adult female breeders were placed on either CHD (control house-chow diet) or HFD (high-fat diet) 4 weeks prior to mating and continued through gestation and lactation. Offspring were weaned on to CHD at postnatal day (PND) 21. On PND90, offspring were injected with corticosterone (CORT), lipopolysaccharide (LPS), or CORT + LPS combination, and sacrificed 3 h later

All litters were culled to 12 pups/litter at postnatal day (PND) 1 (*n* = 6 females and *n* = 6 males where possible) to standardize the degree of maternal care received across litters. Litters were weighed weekly during cage changes and were otherwise left undisturbed until weaning (PND21), when they were all placed on a CHD diet and housed in same-sex pairs. At adulthood (PND90), body weights were measured prior to CORT and LPS injections (Fig. 1). *n* = 1 female and *n* = 1 male offspring were used per litter across four conditions that included: (1) baseline diet (*n* = 6 CHD and *n* = 6 HFD), (2) diet + CORT (*n* = 6 CHD CORT and *n* = 6 HFD CORT), (3) diet + LPS challenge (*n* = 6 CHD LPS and *n* = 6 HFD LPS), and (4) diet + CORT + LPS challenge (*n* = 6 CHD CORT + LPS and *n* = 6 HFD CORT + LPS). In total, 96 female and male offspring at PND90 from *n* = 12 separate litters (*n* = 6 CHD dams and *n* = 6 HFD dams) were used for this study.

### Adult CORT and LPS challenge

CORT dissolved in propylene glycol (27840; Sigma-Aldrich) and LPS from *Escherichia coli* O111:B4, (L2630; Sigma-Aldrich) were used for subcutaneous and intraperitoneal injections, respectively. Adult female and male offspring were handled 2 min/day for five consecutive days starting from PND85. At PND90, animals were divided into one of four experimental groups: (1) subcutaneous dose of CORT (10 mg/mL in polypropylene glycol per kilogram of body weight), (2) an intraperitoneal dose of LPS (50 μg/mL in saline per kilogram of body weight), (3) a simultaneous dose of CORT and LPS (10 mg/mL, 50 μg/mL), or (4) handled, non-injected controls (*n* = 6 per diet, sex, and treatment) [37]. A 10 mg/mL dose of CORT was previously shown to lead to heightened anxiety-like behavior and circulating plasma CORT levels similar to the levels of CORT exhibited after several hours of acute physiological stress exposure [38]. The 50 μg/mL dose of LPS was shown to activate the HPA axis within 0.5–4 h, as shown by increased CORT levels in whole-blood [39] and induce pro-inflammatory cytokine expression in the hippocampus of offspring exposed to maternal HFD [30]. All animals were sacrificed 3 h post injection by CO_2_ inhalation followed by rapid decapitation at the mid-point of the light phase (11am–3pm) of the circadian cycle. The mid-point of the circadian cycle was chosen to minimize the confounding circadian effects on hormone levels (e.g., CORT), behavior, and gene expression [27, 28]. Brains were dissected, flash-frozen in isopentane on dry ice, and stored at – 80 °C for later use.

### RNA extraction and cDNA synthesis

Whole amygdala, dorsal hippocampus, and medial prefrontal cortex were cryosectioned using a Leica CM3050 cryostat and stereotaxic coordinates [40]. RNA was extracted from the amygdala, dorsal hippocampus, and medial prefrontal cortex using TRIzol reagent (15596026; Invitrogen) in combination with RNeasy Plus Mini Kit (74134; Qiagen) as per the manufacturer’s instructions. RNA quantification and quality assessments were done using a Nanodrop Spectrophotometer (ND-2000C; Thermo Scientific). One microgram of total RNA was converted to cDNA using High Capacity cDNA Reverse Transcription Kit (4368814; Applied Biosystems) according to the manufacturer’s instructions.

### Gene expression analysis by qPCR

Relative mRNA expression of glucocorticoid receptor (GR), mineralocorticoid receptor (MR), nuclear factor kappa light chain enhancer of activated B cells (NFκB), nuclear factor of kappa light polypeptide gene enhancer in B cells inhibitor, alpha (IκBα), interleukin 6 (IL6), interleukin 10 (IL10), cluster of differentiation molecule 11B (CD11B), mitogen-activated protein kinase phosphatase 1 (MKP1), and insulin-like growth factor 1 (IGF1) were quantified across three brain regions using a StepOne Plus real-time thermocycler with Fast SYBR Green PCR master mix (4385612; Applied Biosystems). Primers were purchased from Qiagen or Eurofins Genomics and designed according to GenBank sequence information at the National Center for Biotechnology Information (NCBI) (Table 1).

**Table 1:** Primers used in qPCR analysis

Primer	Sequence
*Reference genes*	
β-Actin	FW: TTTGAGACCTTCAACACCCC
	RV: ATAGCTCTTCTCCAGGGAGG
GAPDH	FW: ACATCAAATGGGGTGATGCT
	RV: GTGGTTCACACCCATCACAA
18S rRNA	FW: ATGGTAGTCGCCGTGCCTA
	RV: CTGCTGCCTTCCTTGGATG
YWHAZ	FW: TTGAGCAGAAGACGGAAGGT
	RV: GAAGCATTGGGGATCAAGAA
*Target genes*	
GR	RT^2^ qPCR Primer Assay (PPR52805B, Qiagen)
MR	FW: GGCAGCTGCAAAGTCTTCTT
	RV: GACAGTTCTTTCGCCGAATC
NFκB	RT^2^ qPCR Primer Assay (PPR42746A, Qiagen)
IκBα	FW: CAGGATTCTGCAGGTCCACT
	RV: TGGAGCACTTGGTGACTTTG
IL6	RT^2^ qPCR Primer Assay (PPR06483B, Qiagen)
IL10	RT^2^ qPCR Primer Assay (PPR06479A, Qiagen)
CD11B	FW: GAAGCCTTGGCGTGTGATAG
	RV: GAGCAGTTTGTTCCCAAGGG
MKP1	FW: GCTCCACTCAAGTCTTCTTCCTCCAA
	RV: TGGACTGTTTGCTGCACAGCTCAG
IGF1	FW: GCTCTTCAGTTCGTGTGTGG
	RV: TGAGTCTTGGGCATGTCAGT

Relative gene expression was calculated using the quantity mean based on a standard curve of 11 serial dilutions ranging from 500 ng/μL to 0.49 ng/μL of cDNA. A standard curve was run per plate and per set of comparisons. Quantity means were normalized against the GEOmean of four reference genes, YWAZ, GAPDH, 18S, and Actin B. Reference genes were identified as stable internal controls based on geNORM analysis of stability across experimental groups, brain regions, and sex [41]. Stability *M* values calculated by geNORM: YWAZ = 0.322, Actin B = 0.49, GAPDH = 0.49, 18S =0.68. Relative transcript levels were expressed as mean ± SEM representing *n* = 6 biological replicates per experimental condition.

### Statistical analysis

Data analysis was carried out using SPSS (IBM Corp.) and R Statistical Software (R Foundation for Statistical Computing, Vienna, Austria, 3.4.2). Adult offspring bodyweight was analyzed by 3-way (diet × drug × sex) ANOVA. qPCR data were analyzed within sex and brain region. A Shapiro-Wilk test was used to assess normality for all transcript data as the *n* < 30. All transcript data were normally distributed and as such parametric analyses were carried out. Outliers were examined using boxplots and only extreme outliers with interquartile range of 3 or more were removed from the dataset. General linear model (GLM) univariate analysis was used to test for main effects of diet, challenge, and diet × challenge interactions. The Scheffe post hoc test was used to conduct mean pairwise comparisons between diet and challenge groups. Relationships were considered statistically significant at *p* ≤ 0.05.

## Results

### Offspring body weight

**Fig. 2:**
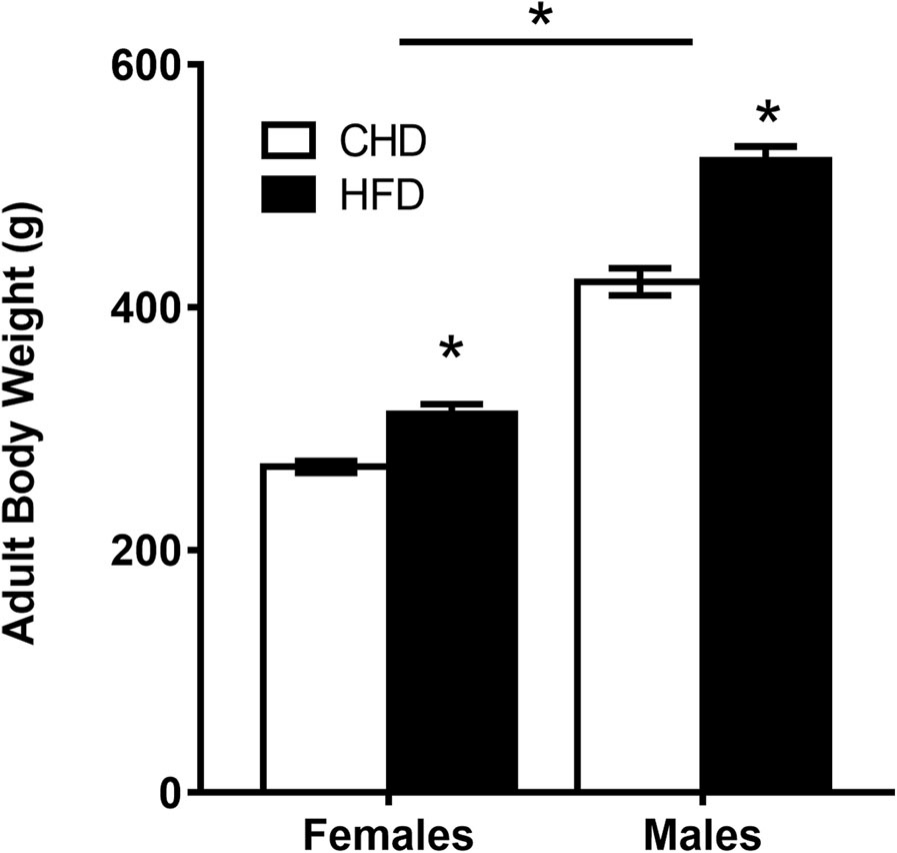
Offspring body weight in adulthood. Average body weight for PND90 offspring per sex and maternal diet condition ± SEM. *n* = 24 animals in total were used per diet group and per sex (*n* = 24 CHD females; *n* = 24 HFD females; *n* = 24 CHD males; *n* = 24 HFD males). *n* = 1 female and *n* = 1 male offspring were sampled per litter across four challenge conditions (baseline diet (*n* = 6), diet + CORT (*n* = 6), diet + LPS (*n* = 6), and diet + CORT + LPS (*n* = 6). All CHD female and male offspring originated from *n* = 6 dams that were on a CHD diet and all HFD female and male offspring originated from *n* = 6 dams that were on a HFD diet. CHD = control house-chow diet, HFD = high-fat diet. *p < 0.0001 for main effect of diet and main effect of sex

The offspring used in this study were littermates of the subjects used in a previously published study from our group [27]. Maternal HFD exposure did not affect offspring body weight at birth; however, offspring born to HFD dams weighed more than offspring born to CHD dams at weaning (PND21) [27]. In adulthood (PND90), male offspring weighed more than female offspring (main effect of sex (*F*_(1,93)_ = 220.20, *p <* 0.001, Fig. 2), and both sexes with maternal HFD exposure weighed more than CHD offspring (females (*F*_(1,46)_ = 19.71, *p* < 0.001), males (*F*_(1,45)_ = 39.82, *p* < 0.01, Fig. 2)). There were no significant differences in body weight between animals assigned across challenge groups (CORT, LPS, CORT + LPS, control handled) at PND90 (data not shown).

### Transcript response to endocrine and immune challenge

#### CORT challenge

**Fig. 3:**
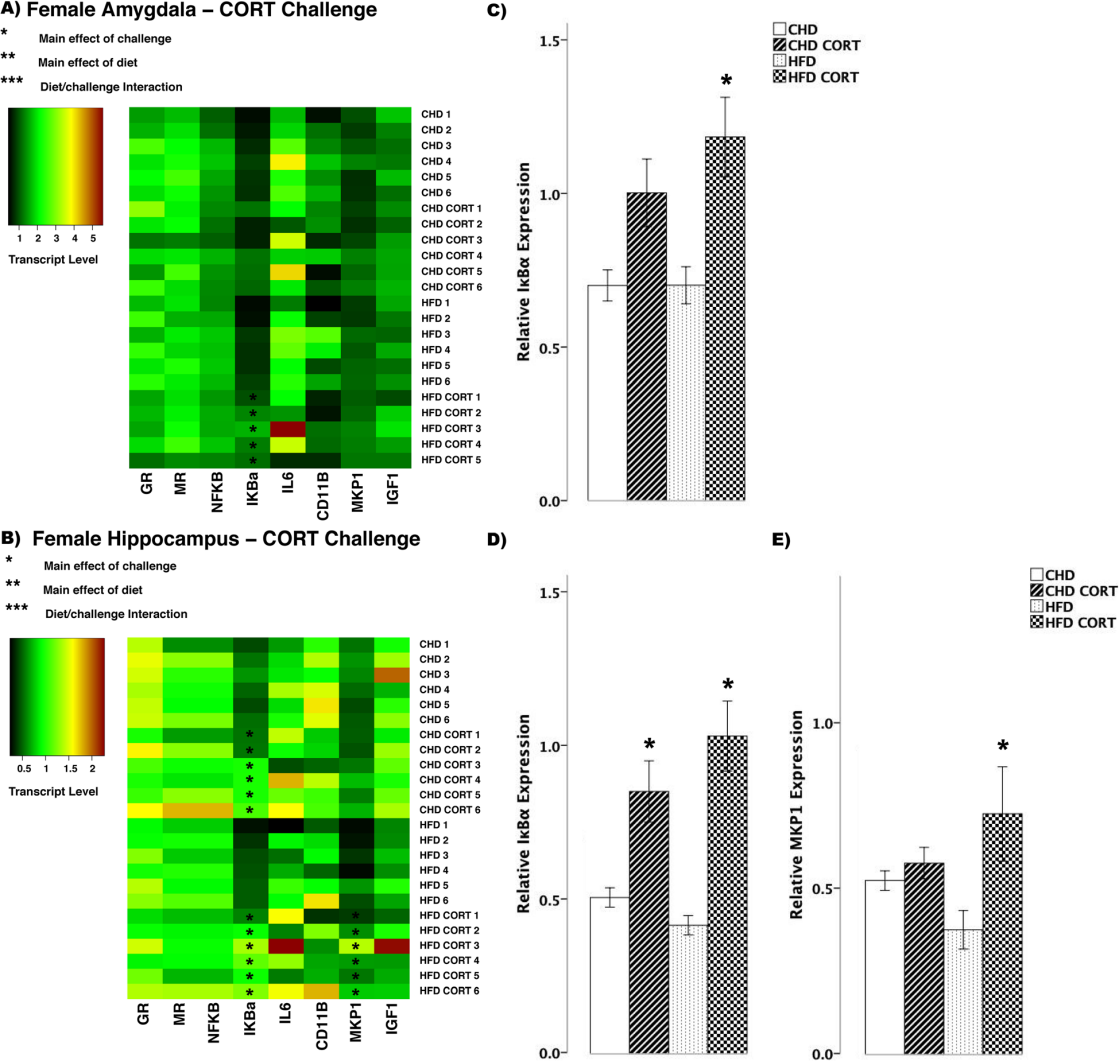
Transcript response to corticosterone (CORT) in the amygdala and hippocampus of adult females. **a, b** Heatmaps represent transcript levels in the amygdala and hippocampus for individual animals. **c** Relative transcript abundance of IκBα (nuclear factor of kappa light polypeptide gene enhancer in B cells inhibitor, alpha) in the amygdala. **d, e** Relative transcript abundance of IκBα, and MKP1 (mitogen activated protein kinase phosphatase 1) in the hippocampus. Data presented are means ± standard error. *n* = 6/experimental group. Main effect of challenge: *p < 0.05. Main effect of diet: **p < 0.05. Diet/challenge interaction: ***p < 0.05. Scheffe post hoc testing was used for pairwise comparisons (*p* < 0.05)

In female offspring, CORT challenge led to few differences in transcript abundance between HFD and CHD offspring (Fig. 3a, b). In the amygdala, IκBα increased in HFD offspring (main effect of challenge (*F*_(3,19)_ = 9.63, *p <* 0.01), Scheffe post hoc *p* = 0.015), but did not change in CHD offspring (Scheffe post hoc *p* = 0.195, Fig. 3c). In the hippocampus, IκBα increased in both diet groups (main effect of challenge (*F*_(3,20)_ = 13.35, *p* < 0.01), Fig. 3d). Also in the hippocampus, MKP1 increased in HFD offspring (main effect of challenge (*F*_(3,20)_ = 12.75, *p* < 0.05), Scheffe post hoc *p* = 0.001), but remained unchanged in CHD offspring (Scheffe post hoc *p* = 0.985, Fig. 3e).

**Fig. 4:**
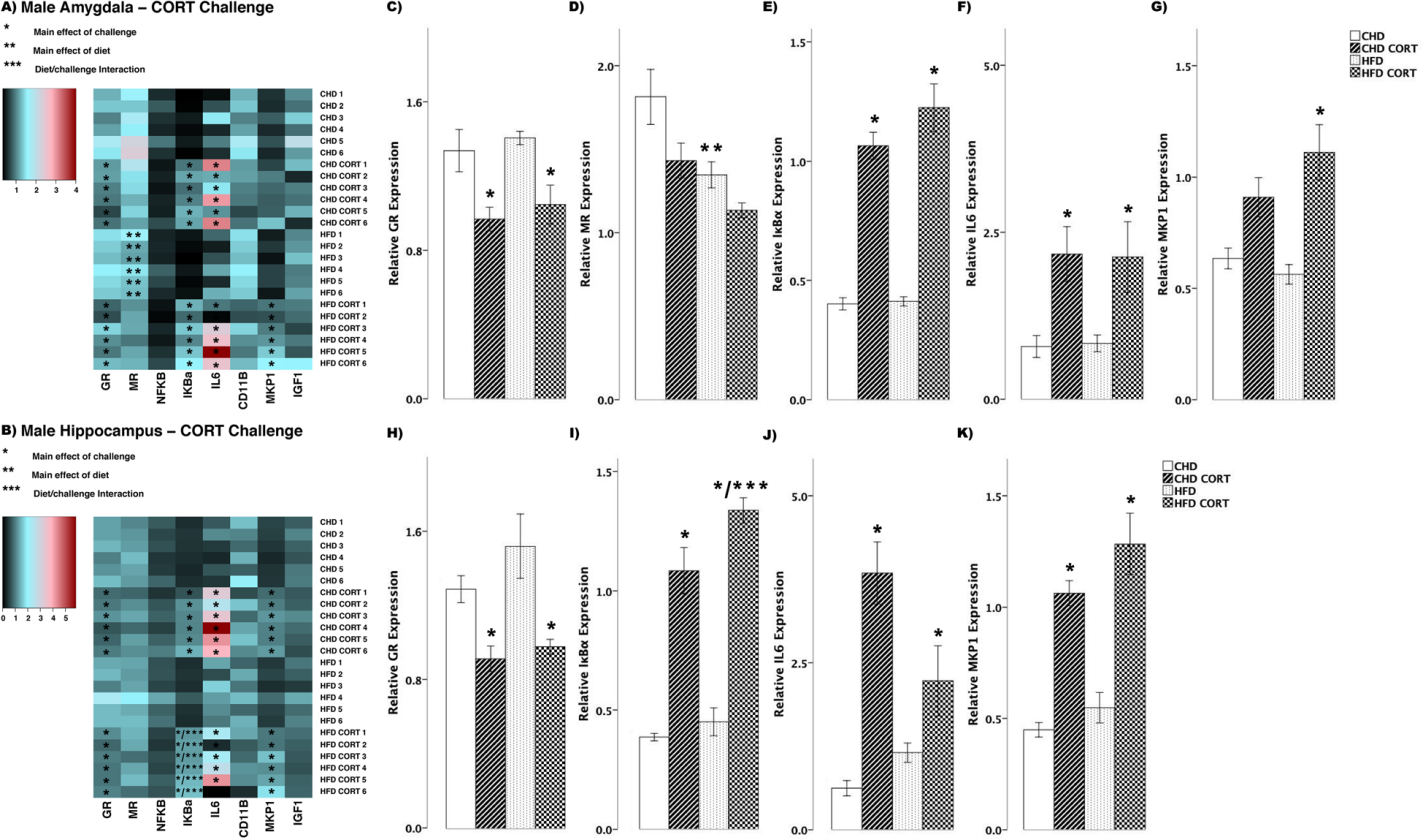
Transcript response to corticosterone (CORT) in the amygdala and hippocampus of adult males. **a, b** Heatmaps represent transcript levels in the amygdala and hippocampus for individual animals. **c–g** Relative transcript abundance of GR (glucocorticoid receptor), MR (mineralocorticoid receptor), IκBα (nuclear factor of kappa light polypeptide gene enhancer in B cells inhibitor, alpha), IL6 (interleukin 6), and MKP1 (mitogen activated protein kinase phosphatase 1) in the amygdala. **h–k** Relative transcript abundance of GR, IκBα, IL6, and MKP1 in the hippocampus. Data presented are means ± standard error. *n* = 6/experimental group. Main effect of challenge: *p < 0.05. Main effect of diet: **p < 0.05. Diet/challenge interaction: ***p < 0.05. Scheffe post hoc testing was used for pairwise comparisons (*p* < 0.05)

In male offspring, we observed more differences in transcript abundance in response to CORT challenge (Fig. 4a, b). In the amygdala, GR decreased in both diet groups (main effect of challenge (*F*_(3,20)_ = 9.006, *p* < 0.01), Fig. 4c), whereas MR decreased in HFD offspring (main effect of diet (*F*_(3,20)_ = 4.147, *p* < 0.01), Scheffe post hoc *p* = 0.047, Fig. 4d). Also in the amygdala, with CORT challenge, IκBα (*F*_(3,20)_ = 52.35, *p* < 0.01, Fig. 4e) and IL6 (*F*_(3,20)_ = 4.82, *p* = 0.01, Fig. 4f) increased in both CHD and HFD groups. MKP1 increased in HFD offspring (main effect of challenge, (*F*_(3,20)_ = 9.46, *p* < 0.01), Scheffe post hoc, *p* = 0.02), but did not change in CHD offspring (Scheffe post hoc, *p* = 0.170, Fig. 4g). In the hippocampus, GR decreased in both diet groups (main effect of challenge, (*F*_(3,20)_ = 7.69, *p* < 0.01), Fig. 4h), whereas IκBα increased in both diet groups (main effect of challenge, (*F*_(3,20)_ = 55.3, *p* < 0.01); diet/challenge interaction (*F*_(3,20)_ = 79.830, *p* < 0.01), Fig. 4i). In both diet groups, CORT challenge led to increases in IL6 (*F*_(3,20)_ = 15.21, *p* < 0.01, Fig. 4j) and MKP1 (*F*_(3,20)_ = 23.41, *p* < 0.01, Fig. 4k) in the hippocampus.

#### LPS challenge

**Fig. 5:**
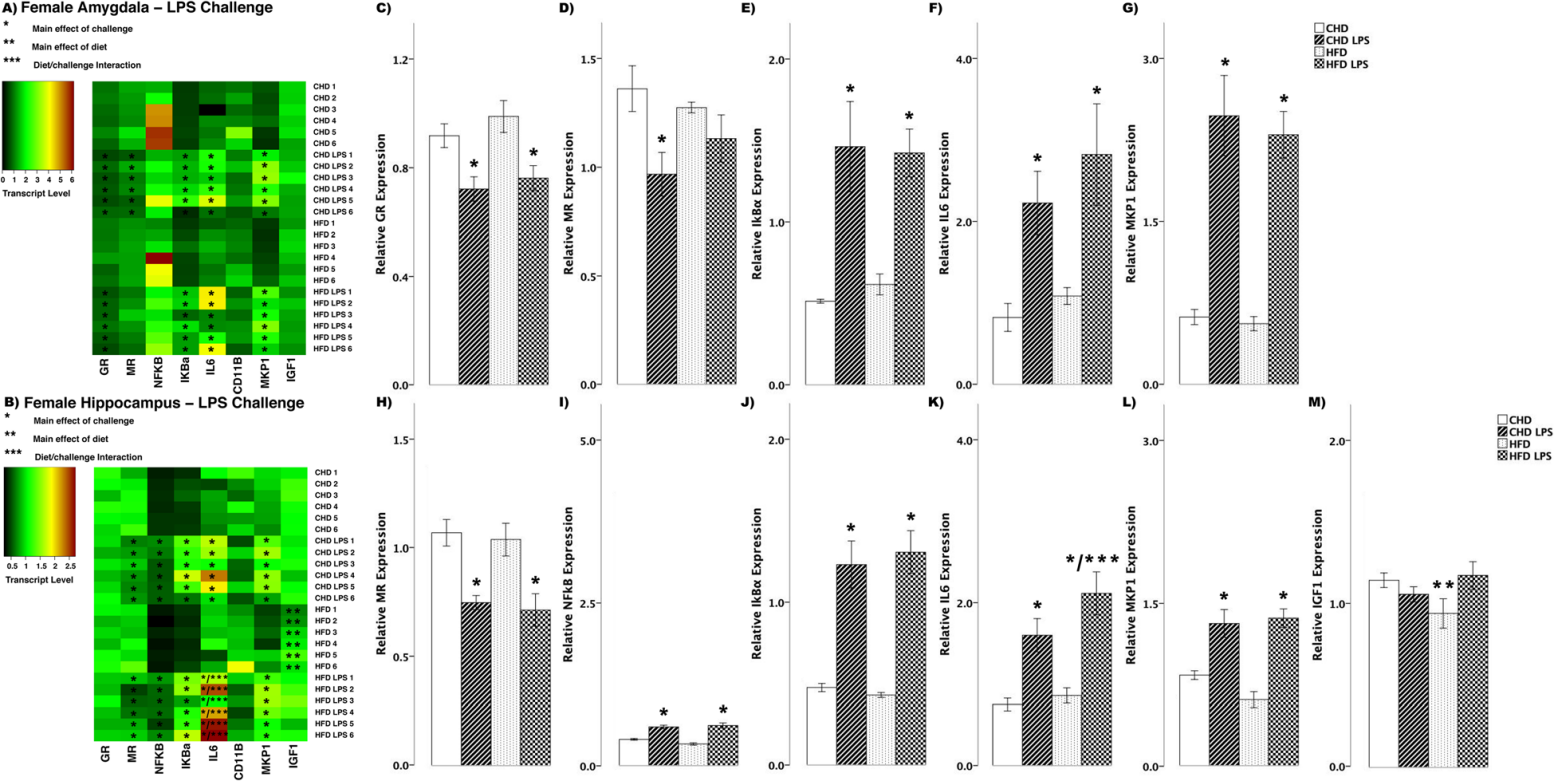
Transcript response to lipopolysaccharide (LPS) in the amygdala and hippocampus of adult females. **a, b** Heatmaps represent transcript levels in the amygdala and hippocampus for individual animals**. c–g** Relative transcript abundance of GR (glucocorticoid receptor), MR (mineralocorticoid receptor), IκBα (nuclear factor of kappa light polypeptide gene enhancer in B cells inhibitor, alpha), IL6 (interleukin 6), and MKP1 (mitogen activated protein kinase phosphatase 1) in the amygdala. **h–m** Relative transcript abundance of MR, NFκB (nuclear factor kappa light chain enhancer of activated B cells), IκBα, IL6, MKP1, and IGF1 (insulin-like growth factor 1) in the hippocampus. Data presented are means ± standard error. *n* = 6/experimental group. Main effect of challenge: *p < 0.05. Main effect of diet: **p < 0.05. Diet/challenge interaction: ***p < 0.05. Scheffe post hoc testing was used for pairwise comparisons (*p* < 0.05)

In female offspring, LPS challenge led to differences in transcript abundance between HFD and CHD offspring (Fig. 5a, b). Both diet groups showed decreased GR in the amygdala (main effect of challenge (*F*_(3,20)_ = 6.74, *p* < 0.01), Fig. 5c). CHD offspring had decreased MR levels (main effect of challenge (*F*_(3,20)_ = 3.55, *p* < 0.01), Scheffe post hoc *p* = 0.04), whereas MR levels in HFD offspring remained unchanged (Scheffe post hoc *p* = 0.749, Fig. 5d). Also in the amygdala of both diet groups, there were increases in IκBα (main effect of challenge (*F*_(3,20)_ = 10.07, *p* < 0.01), Fig. 5e), IL6 (*F*_(3,20)_ = 6.12, *p* < 0.01, Fig. 5F), and MKP1 (*F*_(3,20)_ = 22.63, *p* < 0.01, Fig. 5G). In the hippocampus of both diet groups, LPS challenge led to decreased MR transcript (main effect of challenge (*F*_(3,20)_ = 8.70, *p* < 0.01), Fig. 5h), while there were increases in NFκB (*F*_(3,20)_ = 26.80, *p* < 0.01, Fig. 5i), and IκBα (*F*_(3,20)_ = 23.24, *p* < 0.01, Fig. 5j). IL6 transcript increased in both diet groups, however, there was a larger increase in HFD females (main effect of challenge (*F*_(3,20)_ = 6.12, *p* < 0.01); diet/challenge interaction (*F*_(3,20)_ = 2.75, *p* < 0.05, Fig. 5k)). MKP1 levels also increased in both diet groups in female offspring (main effect of challenge (*F*_(3,20)_ = 17.57, *p* < 0.01), Fig. 5l). IGF1 transcript levels were lower in HFD females when compared to CHD in basal conditions (main effect of diet (*F*_(3, 20)_ = 2.26, *p* < 0.05), Fig. 5m).

**Fig. 6:**
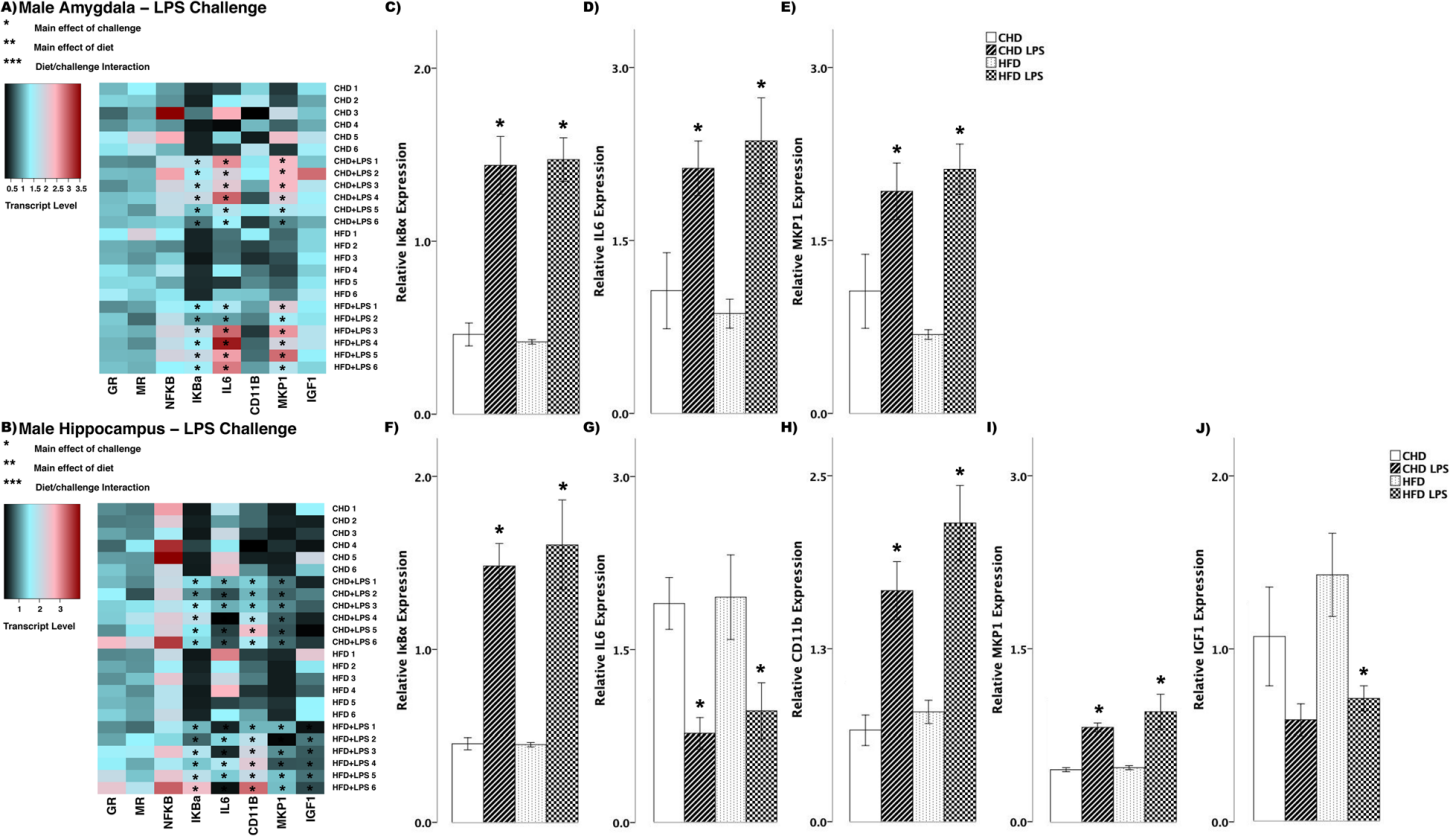
Transcript response to lipopolysaccharide (LPS) in the amygdala and hippocampus of adult males. **a, b** Heatmaps represent transcript levels in the amygdala and hippocampus for individual animals**. c–e** Relative transcript abundance of IκBα (nuclear factor of kappa light polypeptide gene enhancer in B cells inhibitor, alpha), IL6 (interleukin 6), and MKP1 (mitogen activated protein kinase phosphatase 1) in the amygdala. **f–j** Relative transcript abundance of IκBα, IL6, CD11B (cluster of differentiation molecule 11B), MKP1, and IGF1 (insulin-like growth factor 1) in the hippocampus. Data presented are means ± standard error. *n* = 6/experimental group. Main effect of challenge: *p < 0.05. Main effect of diet: **p < 0.05. Diet/challenge interaction: ***p < 0.05. Scheffe post hoc testing was used for pairwise comparisons (*p* < 0.05)

In male offspring, there were fewer differences in transcript abundance between CHD and HFD groups in response to LPS challenge (Fig. 6a, b). In the amygdala of both diet groups, there were increases in IκBα (main effect of challenge (*F*_(3,20)_ = 28.29, *p* < 0.01), Fig. 6c), IL6 (*F*_(3,20)_ = 7.09, *p* < 0.01, Fig. 6d), and MKP1 (*F*_(3,20)_ = 8.84, *p* < 0.01, Fig. 6e) in response to LPS challenge. Similar to changes in the amygdala, in the hippocampus of both diet groups, LPS challenge led to increases in IκBα (main effect of challenge (*F*_(3,20)_ = 18.79, *p* < 0.01), Fig. 6f), while IL6 levels decreased in both diet groups (*F*_(3,20)_ = 5.76, *p* < 0.01, Fig. 6g). CD11B (main effect of challenge (*F*_(3,20)_ = 14.95, *p* < 0.01), Fig. 6h) and MKP1 (*F*_(3,20)_ = 9.98, *p* < 0.01, Fig. 6i) levels increased in both diet groups in male offspring. Lastly, IGF1 expression decreased in HFD males (main effect of challenge (*F*_(3,20)_ = 3.73, *p* < 0.05), Scheffe post hoc *p* = 0.017), but remained unchanged in CHD counterparts (Scheffe post hoc *p* = 0.410, Fig. 6j).

#### Combined CORT and LPS challenge

**Fig. 7:**
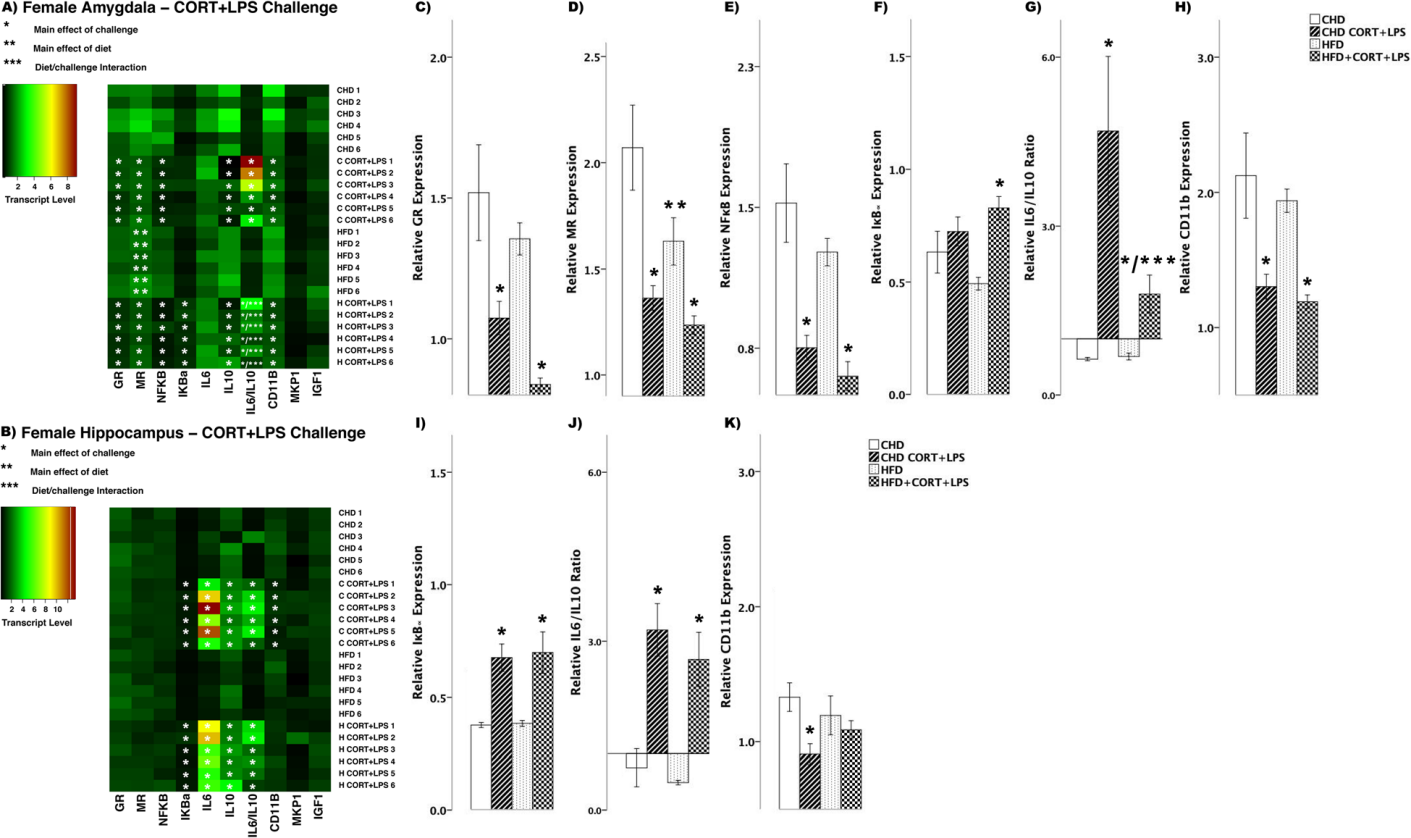
Transcript response to CORT+LPS in the amygdala and hippocampus of adult females. **a, b** Heatmaps represent transcript levels in the amygdala and hippocampus for individual animals**. c–h** Relative transcript abundance of GR (glucocorticoid receptor), MR (mineralocorticoid receptor), NFκB (nuclear factor kappa light chain enhancer of activated B cells), IκBα (nuclear factor of kappa light polypeptide gene enhancer in B cells inhibitor, alpha), the ratio of pro versus anti inflammation designated by IL6/IL10 (interleukin 6/10), and CD11B (cluster of differentiation molecule 11B) expression in the amygdala. **i–k** Relative transcript abundance of IκB, IL6/IL10 ratio, and CD11B in the hippocampus. Data presented are means ± standard error. *n* = 6/experimental group. Main effect of challenge: *p < 0.05. Main effect of diet: **p < 0.05. Diet/challenge interaction: ***p<0.05. Scheffe post-hoc testing was used for pairwise comparisons (*p* < 0.05)

In female offspring, CORT + LPS challenge lead to several differences in transcript abundance between HFD and CHD offspring (Fig. 7a, b). In the amygdala, both diet groups showed decreases in GR (main effect of challenge (*F*_(3,20)_ = 10.0, *p* < 0.01), Fig. 7c), MR (main effect of challenge (*F*_(3,20)_ 9.42, *p* < 0.01); main effect of diet (*F*_(3,20)_ = 5.596, *p* < 0.05), Fig. 7d), and NFκB (main effect of challenge (*F*_(3,20)_ = 12.52, *p* < 0.01), Fig. 7e). In contrast, IκBα levels increased in HFD offspring (main effect of challenge (*F*_(3,20)_ = 4.68, *p* < 0.05), Scheffe post hoc *p* = 0.015), but did not change in CHD offspring (Scheffe post hoc *p* = 0.803, Fig. 7f). The ratio of IL6/IL10 was above 1 in both diet groups in the amygdala of female offspring (main effect of challenge (*F*_(3,20)_ = 9.48, *p* < 0.01); diet/challenge interaction (*F*_(3,20)_ = 4.36, *p* < 0.05), Fig. 7g). Lastly, CD11B levels decreased in both diet groups (main effect of challenge (*F*_(3,20)_ = 7.19, *p* < 0.01), Fig. 7h). In the hippocampus, IκBα (main effect of challenge (*F*_(3,20)_ = 10.13, *p* < 0.01), Fig. 7i) and the IL6/IL10 ratio (*F*_(3,20)_ = 13.06, *p* < 0.01, Fig. 7j), increased in both diet groups, while CD11B decreased in CHD females (main effect of challenge (*F*_(3,20)_ = 4.456, *p* < 0.05), Scheffe post hoc *p* = 0.047) and no change was seen in HFD offspring (Scheffe post-hoc *p* = 0.909, Fig.7k).

**Fig. 8:**
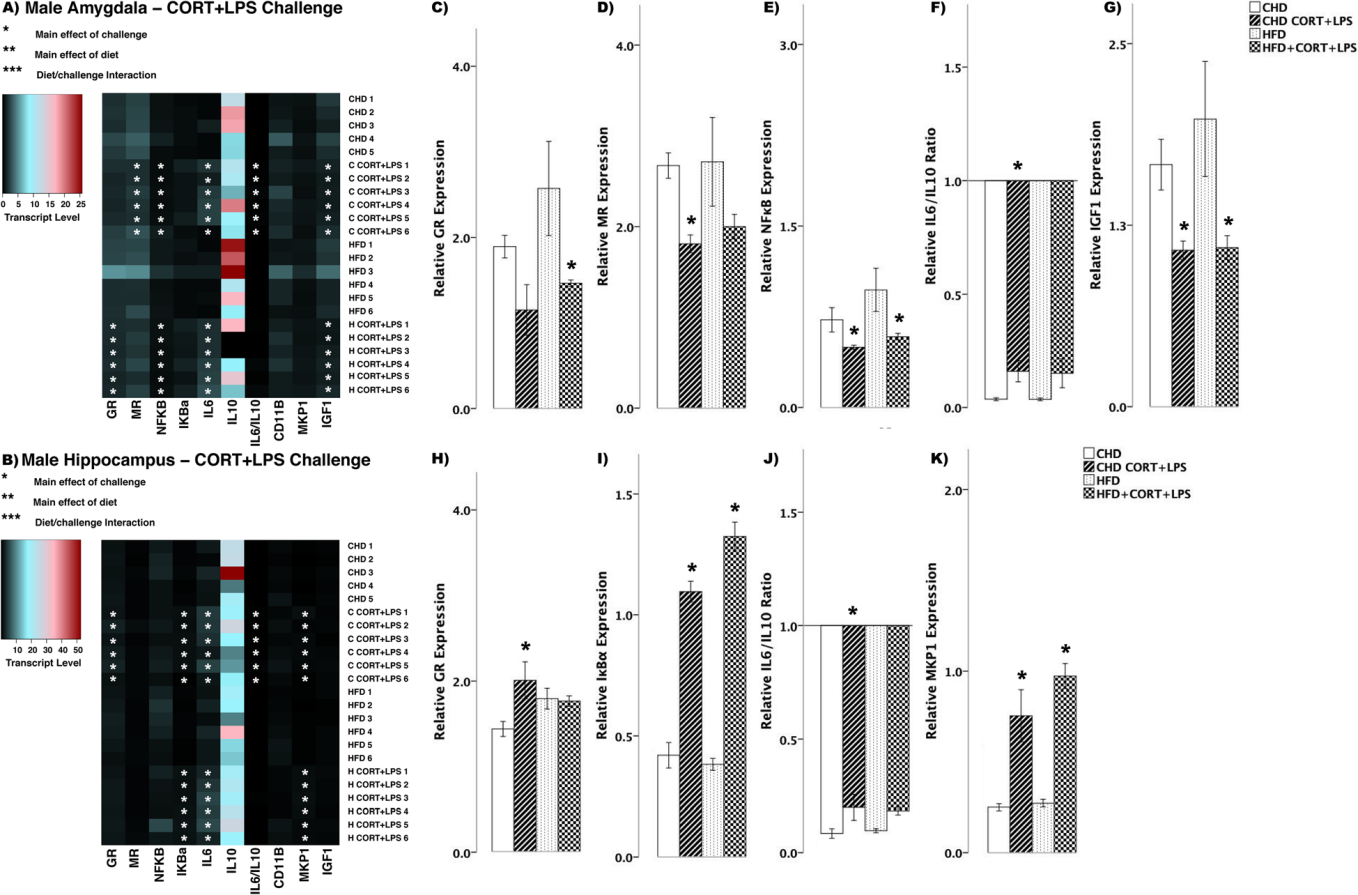
Transcript response to CORT+LPS in the amygdala and hippocampus of adult males. **a, b** Heatmaps represent transcript levels in the amygdala and hippocampus for individual animals**. c–g** Relative transcript abundance of GR (glucocorticoid receptor), MR (mineralocorticoid receptor), NFκB (nuclear factor kappa light chain enhancer of activated B cells), the ratio of pro versus anti inflammation designated by IL6/IL10 (interleukin 6/10), and IGF1 expression in the amygdala. **h–k** Relative transcript abundance of GR, IκBα, IL6/IL10 ratio, and MKP1 in the hippocampus. Data presented are means ± standard error. *n* = 6/experimental group. Main effect of challenge: *p < 0.05. Main effect of diet: **p < 0.05. Diet/challenge interaction: ***p < 0.05. Scheffe post hoc testing was used for pairwise comparisons (*p* < 0.05)

In male offspring, there were fewer differences in transcript abundance between diet groups in response to combined CORT + LPS challenge (Fig. 8a, b). In the amygdala, GR decreased in HFD offspring (main effect of challenge (*F*_(3,20)_ = 4.364, *p* < 0.05), Scheffe post hoc *p* = 0.046), yet remained unchanged in CHD offspring (Scheffe post hoc *p* = 0.512, Fig. 8c). MR levels decreased in CHD offspring (main effect of challenge (*F*_(3,20)_ = 4.110, *p* < 0.05), Scheffe post hoc *p* = 0.022), yet remained unchanged in HFD offspring (Scheffe post hoc *p* = 0.356, Fig. 8d). NFκB levels decreased in both diet groups (main effect of challenge (*F*_(3,20)_ = 4.44, *p* < 0.05), Fig. 8e), where as IL6/IL10 ratio decreased in CHD males (main effect of challenge (*F*_(3,20)_ = 4.458, *p*<0.05), Scheffe post hoc *p* = 0.024), but remained unchanged in HFD offspring (Scheffe post hoc *p* = 0.276, Fig. 8f). Lastly, IGF1 levels decreased in both groups in response to CORT + LPS challenge (*F*_(3,20)_ = 4.29, *p* < 0.05, Fig. 8g). In the hippocampus, there was increased GR levels in CHD offspring (main effect of challenge (*F*_(3,20)_ = 4.90, *p* < 0.05), Scheffe post hoc *p* = 0.044), yet remained unchanged in HFD offspring (Scheffe post hoc *p* = 0.999, Fig. 8h). IκBα transcript levels increased in both diet groups (main effect of challenge (*F*_(3,20)_ = 19.08, *p* < 0.01), Fig. 8i). Similar to that of GR, IL6/IL10 ratio increased in CHD offspring (main effect of challenge (*F*_(3,20)_ = 5.107, *p* < 0.05), Scheffe post hoc *p* = 0.025) and did not change in HFD counterparts (Scheffe post hoc, *p* = 0.319, Fig. 8j). Lastly, MKP1 levels increased in both groups (main effect of challenge (*F*_(3,20)_ = 15.42, *p* < 0.01), Fig. 8k) in response to CORT + LPS challenge.

**Fig. 9:**
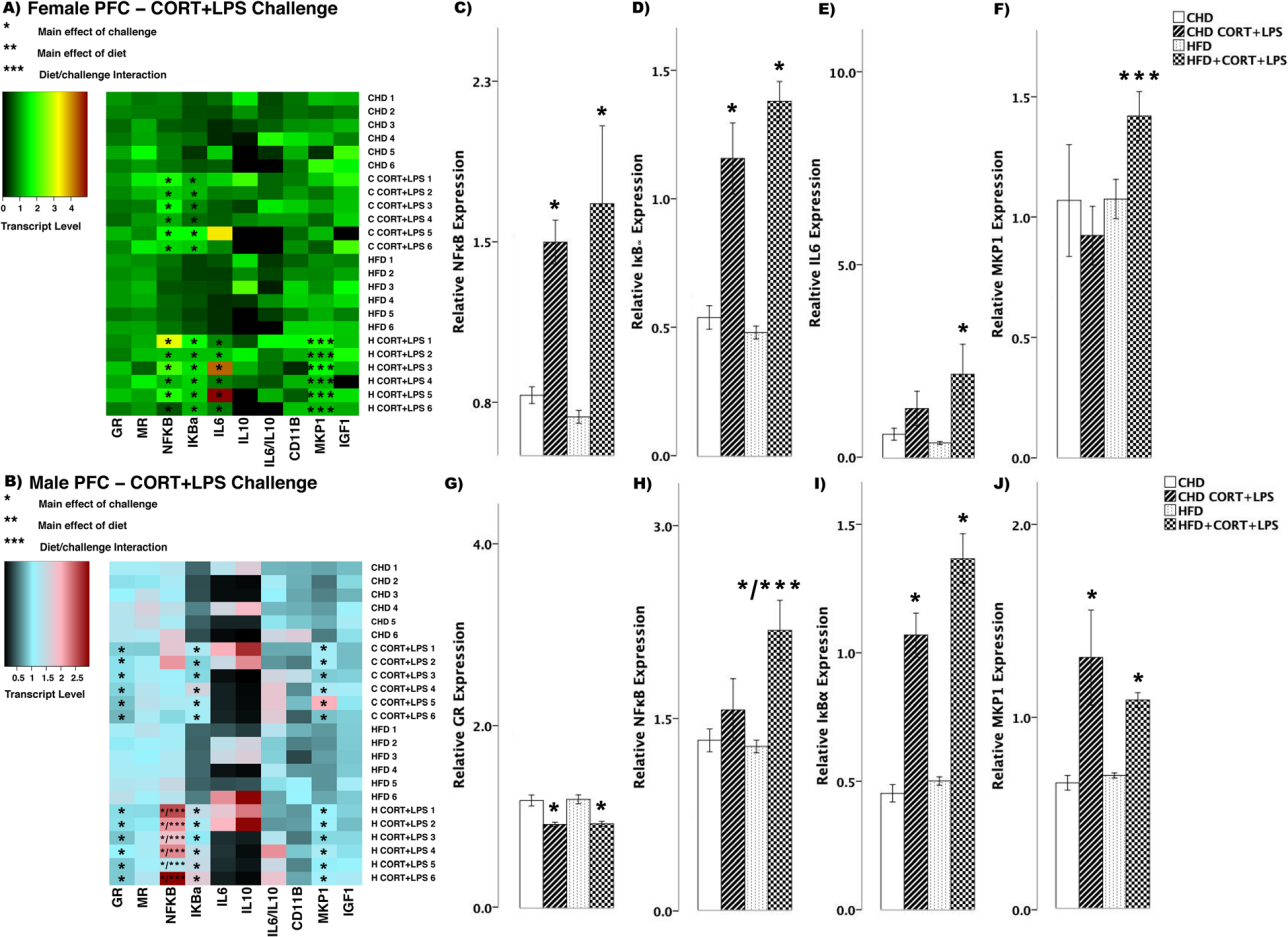
Transcript response to CORT+LPS in the prefrontal cortex of adult females and males. **a, b** Heatmaps represent transcript levels in the PFC for individual female and male animals**. c–f** Relative transcript abundance of NFκB (nuclear factor kappa light chain enhancer of activated B cells), IκBα (nuclear factor of kappa light polypeptide gene enhancer in B cells inhibitor, alpha), IL6 (interleukin 6), and MKP1 (mitogen activated protein kinase phosphatase 1) in the PFC of females. **g–j** Relative transcript abundance of GR, NFκB, IκBα, and MKP1 in the PFC of males. Data presented are means ± standard error. *n* = 6/experimental group. Main effect of challenge: *p < 0.05. Main effect of diet: **p < 0.05. Diet/challenge interaction: ***p < 0.05. Scheffe post hoc testing was used for pairwise comparisons (*p* < 0.05)

To better characterize the neural transcript response to combined CORT and LPS exposure, an additional brain region regulating the HPA axis was assessed; the medial prefrontal cortex (PFC; Fig. 9a, b). In female offspring in response to simultaneous CORT + LPS challenge, both diet groups showed increased levels of NFκB (main effect of challenge (*F*_(3,20)_ = 6.85, *p* < 0.01), Fig. 9c) and IκBα (*F*_(3,20)_ = 28.95, *p* < 0.01, Fig. 9d), while IL6 increased in HFD offspring (main effect of challenge (*F*_(3,20_ = 4.347, *p* < 0.05), Scheffe post hoc *p* = 0.040), but did not change in CHD offspring (Scheffe post hoc *p* = 0.784, Fig. 9e). MKP1 levels increased in HFD offspring with CORT + LPS challenge (diet/challenge interaction (*F*_(3,20)_ = 2.08, *p* < 0.05), Fig. 9f) when compared to CHD counterparts.

In male offspring, GR levels decreased in the PFC of both diet groups (*F*_(3,20)_ = 13.45, *p* < 0.01, Fig. 9g) in response to combined CORT and LPS treatment. NFκB levels increased in HFD males (main effect of challenge (*F*_(3,20)_ = 8.111, *p* < 0.01), Scheffe post hoc *p* = 0.007); diet/challenge interaction (*F*_(3,20)_ = 5.486, *p* < 0.01), whereas no change was seen in CHD offspring (Scheffe post hoc *p* = 0.994, Fig. 9h). Lastly, CORT + LPS challenge led to increased IκBα (*F*_(3,20)_ = 42.56, *p* < 0.01, Fig. 9i) and MKP1 (*F*_(3,20)_ = 9.53, *p* < 0.01, Fig. 9j) levels in both CHD and HFD male offspring.

## Discussion

**Fig. 10:**
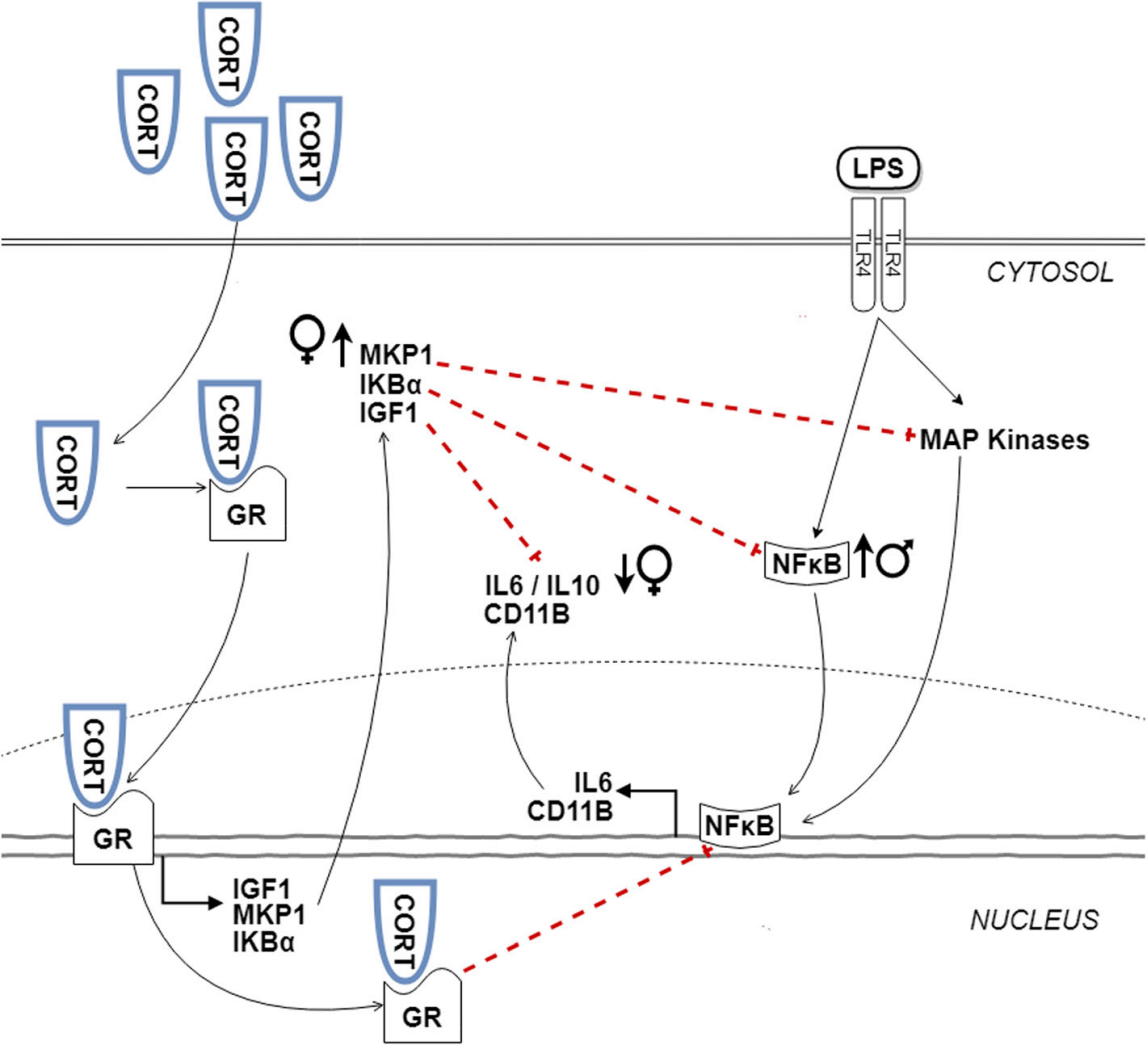
Maternal high-fat diet induces sex-specific effects on transcript responses to CORT + LPS challenge in adulthood. CORT diffuses into the cytosol and binds to glucocorticoid receptor (GR). The CORT-GR complex facilitates the expression of anti-inflammatory molecules including IGF1, MKP1, and I κBα, which at basal levels inhibit pro-inflammatory expression of NFκB, IL6, and CD11B induced by LPS signalling to toll-like receptor 4 (TLR4) and mitogen-activated protein (MAP) kinases. However, with higher levels of CORT, anti-inflammatory effects are reduced, leading to an overall increase in pro-inflammation. In the current study, females exhibited increased MKP1 and a lower IL6/IL10 ratio, while males showed increased NFκB transcript in response to maternal HFD (indicated by arrows)

Overall, acute CORT administration further exaggerated pro-inflammatory responses induced by LPS in both males and females in the combination challenge. However, perinatal HFD programming lead to higher levels of pro-inflammation in males, while females exhibited elevated levels of anti-inflammatory markers (Fig. 10). These findings indicate distinct transcriptional responses to elevated CORT and LPS among HFD-exposed offspring, some of which are sex-specific.

### Enhanced anti-inflammatory responses to CORT challenge in male HFD offspring

CORT is classically associated with anti-inflammatory and immunosuppressive effects. The anti-inflammatory actions of CORT include increased expression of IGF1, IκBα, and MKP1 [11, 42]. With these genes induced, CORT not only acts genomically, but also communicates with kinase pathways and transcription factors. For example, MKP1 is induced in situations of cellular stress and inactivates mitogen-activated protein (MAP) kinases including extracellular-regulated kinases (ERKs), c-Jun-N-terminal kinases (JNKs), stress-activated protein kinases (SAPK), and p38. Inactivation of these kinases inhibits pro-inflammatory signalling that normally leads to NFκB activation. IκBα binds to the p65 subunit of NFκB and prevents activation and nuclear translocation of NFκB. In addition, IκBα promotes the expression of anti-inflammatory cytokines, including IL-10 [43–47].

In the hippocampus, both diet groups and sexes showed increased expression of IκBα in response to CORT challenge (Figs. 3 and 4). This was expected, as CORT binds to GR in the cytosol and translocates to the nucleus and promote anti-inflammatory gene expression, including IκBα [3, 4, 11]. However, in males, the increase in IκBα in response to CORT was greater in HFD offspring compared to CHD offspring. Moreover, HFD males showed increased IκBα levels compared to CHD males in basal conditions. This may be an adaptation to developmental HFD exposure, as HFD males also showed elevated levels of the pro-inflammatory cytokine, IL6, in basal conditions. Overall, in the amygdala, HFD males showed reduced MR transcript in response to CORT compared to CHD males. It is possible that reduced MR transcript in HFD males may have further potentiated GR-induced anti-inflammation, as reduced availability of MR and receptor saturation with CORT is associated with increased GR expression [11, 48, 49]. However, it is unclear whether CORT binding to MR would lead to pro- or anti-inflammatory gene expression, as the functional roles of MR in modulating inflammation is not well understood [10]. A study using in vitro murine BV-2 microglial cells reported that MR exerts pro-inflammatory effects by potentiating the expression of IL6, tumour necrosis factor-alpha (TNFα), and NFκB activation in the presence of CORT, whereas GR suppressed the expression of these pro-inflammatory mediators [50]. Although further in vitro and in vivo studies are required to analyze the effects of MR in mediating inflammation, these findings suggest that male offspring exposed to maternal HFD may tolerate acute psychological or endocrine stressors more efficiently than CHD offspring in adulthood, initiating protective, anti-inflammatory signalling through enhanced IκBα expression in the hippocampus, and reduced MR expression in the amygdala.

### Enhanced pro-inflammatory response to LPS challenge in female HFD offspring

LPS binds to toll-like receptor 4 (TLR4) and activates MAP kinase pathways including ERK, JNK, p38, and SAPK. The overall activation of MAPK pathways leads to the activation and nuclear translocation of p65 subunit of NFκB, enhances transcription factor binding of activator protein-1 (AP-1), and promotes the expression of cAMP response element-binding protein (CREB), mitogenic, and pro-inflammatory regulators [43–47]. LPS-TLR4 signalling further promotes pro-inflammatory responses by activating IκB kinases (IKK), which phosphorylates IκBα and prevents IκBα:NFκB heterodimerization. Unbound NFκB is then free to translocate to the nucleus and induce expression of pro-inflammatory cytokines, including IL6 [43–47]. Over time, pro-inflammatory activation of the HPA axis leads to a homeostatic state through CORT-GR-mediated expression of anti-inflammatory genes, including MKP1 and IκBα, which prevent further accumulation of cytotoxic pro-inflammatory cytokines [1, 51–55].

As expected, LPS challenge led to increases in both pro- and anti-inflammatory transcripts in both sexes and diet groups, including IL6, IκBα, and MKP1. However, in the hippocampus there was a potentiation of NFκB and IL6 transcript response in HFD females exposed to LPS challenge (Figs. 5 and 6). This pro-inflammatory phenotype has been observed in previous studies, where rats exposed to maternal HFD exhibited elevated levels of TLR4 at PND 0, as well as larger pro-inflammatory cytokine responses in peripheral blood in adulthood [30]. Thus, the enhanced inflammation observed in this study may result from persistent and enhanced TLR4 expression in the brain of adult offspring exposed to maternal HFD, though this remains to be examined. Moreover, chronic HFD consumption leads to increased inflammation and neuronal apoptosis in the hypothalamus [44, 56]. It is possible that exacerbated inflammation-induced apoptosis may occur in other regions of the brain, including the hippocampus, in offspring exposed to maternal HFD, altering neuronal circuitry that governs HPA axis and CORT secretion, though this remains to be examined.

### Sex-specific alterations to CORT+LPS challenge in HFD offspring

Basal levels of CORT are known to dampen pro-inflammatory effects of LPS through GC signalling and anti-inflammatory gene expression. A previous study reported acute elevations in CORT through exogenous administration or psychosocial stress leads to increased pro-inflammatory transcript expression and decreased anti-inflammatory transcript expression [3, 5]. A mechanism for this action could be that with increases in CORT levels, there is increased ERK, JNK, and p38 kinase activation, as observed in previous studies in frontal cortex and hippocampus, even in the absence of inflammatory triggers such as LPS [3].

In our current study, CORT and LPS exposure in the amygdala of female offspring exposed to maternal HFD led to a lower pro-/anti-inflammatory IL6/IL10 cytokine transcript ratio relative to CHD females, indicating an enhanced anti-inflammatory transcriptional response (Fig. 7). In parallel, only HFD females displayed an increase in MKP1 (Fig. 9), an anti-inflammatory marker, in the PFC. These findings suggest that maternal HFD programming may be associated with increased anti-inflammatory transcriptional response in the amygdala and PFC of females potentially as an adaptive response to neuroinflammatory damage. Our study focused on IL10 expression as a marker of anti-inflammation; however, there are alternate anti-inflammatory cytokines that may also be involved in the CORT+LPS signalling, including IL4 and IL13 [57]. Further investigation is warranted to understand the interplay between pro- and anti-inflammation in limbic brain regions in response to combined CORT and LPS challenge. Furthermore, CORT levels were not measured in this study; however, a separate study with littermates of the current subjects indicated that adult rats with maternal HFD exposure exhibit lower levels of basal CORT compared to CHD offspring [27]. In particular, female HFD offspring, continued to have lower stress-induced CORT after a 20-min restraint challenge [27] Therefore, an acute administration of exogenous CORT to the periphery during adulthood may not be sufficient to elicit a potentiation of pro-inflammation due to the lower levels of basal CORT present in HFD offspring.

In stark contrast to females, simultaneous CORT and LPS exposure in males led to minimal differences in the amygdala and hippocampus between the diet groups (Fig. 8). However, in the PFC, CORT, and LPS exposure led to a significant increase in NFκB in HFD males (Fig. 9). These findings suggest that in contrast to female offspring, maternal HFD programming may exacerbate NFκB-mediated pro-inflammatory transcriptional responses to combined CORT and LPS challenge in the PFC of adult males. These sex-specific changes in gene expression support previous findings indicating that underlying differences between the female and male brain contribute to distinct inflammatory responses, including those resulting from exposure to gonadal steroid hormones that differentially affect brain development, structure, and function [58, 59].

It is tempting to speculate that these enhanced pro-inflammatory transcript responses may arise as an adaptation to perinatal stress induced by maternal HFD exposure [60]. In the context of maternal obesity induced by high levels of saturated fat, elevations in circulating fat and adiposity are known to stimulate inflammation, which in turn promotes CORT elevations through HPA axis activation [61, 62]. In the mother, elevated CORT and inflammatory molecules can be transmitted to developing offspring both prenatally and during lactation [63–65]. It is possible that exposures to elevated levels of CORT and inflammation during these critical points in development program HPA axis activity and glucocorticoid signalling in offspring, enabling adaptation to chronic inflammation experienced in perinatal life [27–33]. Inasmuch as these alterations rendered by perinatal cues of inflammation do not match the postnatal environment, they may constitute a developmental mechanism leading to phenotypic changes in behavior later in life. Consistent with this hypothesis, elevated anxiety behavior is associated with pro-inflammatory gene expression and inflammation in brain regions regulating the HPA axis, including the amygdala, hippocampus, and PFC [66, 67]. In this study, the hippocampus was the site of enhanced anti-inflammatory responses post-CORT in HFD males and pronounced pro-inflammatory responses post-LPS in HFD females. With CORT + LPS challenge, males showed enhanced pro-inflammatory responses in the PFC, while females showed the opposite in both the PFC and amygdala. Future studies are necessary to assess how the sex- and brain region-specific alterations to the inflammatory transcript response to CORT and LPS observed in the present study alter anxiety-like behavior in offspring with perinatal HFD exposure. In addition, although beyond the scope of the present investigation, we recognize that a comprehensive examination of GR phosphorylation sites and their mutual interactions is needed to fully examine GR regulation.

## Conclusion

In this study, we found that maternal HFD exposure altered transcript responses in adult offspring, through enhanced anti-inflammation post-CORT challenge in males and enhanced pro-inflammation post-LPS in females. We also found evidence of enhanced pro-inflammation post-CORT + LPS challenge in males, with females exhibiting enhanced anti-inflammation. Our findings suggest that exposure to maternal HFD during early life induces sex-specific transcriptional responses to stress in adulthood. It is currently unknown whether humans exposed to maternal obesity and high-fat diets may show similar responses to glucocorticoid and immune stress. However, past studies in rodents and humans indicating that obesity leads to prolonged inflammation and increased sensitivity to infection, suggesting that these effects should also be examined in the context of humans exposed to obesity during development [68–70].

## Data Availability

The data used in this study are available from the corresponding author upon request.

## Abbreviations

ANOVA: Analysis of variance
CD11B: Cluster of differentiation molecule B
CHD: Control house-chow diet
CORT: Corticosterone
GAPDH: Glyceraldehyde 3-phosphate dehydrogenase
HFD: High-fat diet
HPA: Hypothalamic-pituitary-adrenal
HPC: Hippocampus
IGF1: Insulin-like growth factor 1
IκBα: Nuclear factor of kappa light polypeptide gene enhancer in B-cells inhibitor, alpha
IL6: Interleukin 6
IL10: Interleukin 10
GR: Glucocorticoid receptor
LPS: Lipopolysaccharide
MKP1: Mitogen-activated protein kinase phosphatase 1
MR: Mineralocorticoid receptor
NFκB: Nuclear factor kappa-beta-light-chain-enhancer of activated B cells
PFC: Prefrontal cortex
PND: Postnatal day
qPCR: Quantitative polymerase chain reaction
SEM: Standard error of the mean
TNFα: Tumor necrosis factor alpha
YWHAZ: Tyrosine 3-monooxygenase/tryptophan 5-monooxygenase activation protein zeta
